# Practical Pharmacist-Led Interventions to Improve Antimicrobial Stewardship in Ghana, Tanzania, Uganda and Zambia

**DOI:** 10.3390/pharmacy9030124

**Published:** 2021-07-08

**Authors:** Frances Kerr, Israel Abebrese Sefah, Darius Obeng Essah, Alison Cockburn, Daniel Afriyie, Joyce Mahungu, Mariyam Mirfenderesky, Daniel Ankrah, Asiwome Aggor, Scott Barrett, Joseph Brayson, Eva Muro, Peter Benedict, Reem Santos, Rose Kanturegye, Ronald Onegwa, Musa Sekikubo, Fiona Rees, David Banda, Aubrey Chichonyi Kalungia, Luke Alutuli, Enock Chikatula, Diane Ashiru-Oredope

**Affiliations:** 1 Pharmacy, NHS Lanarkshire C/O Monklands Hospital, Airdrie ML6 0JS, UK; frances.kerr@nhs.scot; 2 Pharmacy Department, Keta Municipal Hospital, Keta P.O. Box WT 82, Ghana; isefah1980@gmail.com (I.A.S.); dacode9@gmail.com (D.O.E.); 3 Pharmacy, NHS Lothian, Edinburgh EH1 3EG, UK; Alison.cockburn@nhslothian.scot.nhs.uk; 4 Pharmacy Department, Ghana Police Hospital, Accra P.O. Box CT104, Ghana; dspdan77@yahoo.com; 5 Pharmacy, North Middlesex University Hospital NHS Trust (NMUH), London N18 1QX, UK; Joyce.mahungu@nhs.net; 6 Microbiology, North Middlesex University Hospital NHS Trust (NMUH), London N18 1QX, UK; mariyam.mirfenderesky@nhs.net; 7 Pharmacy Department, Korle-Bu Teaching Hospital (KBTH), Accra P.O. Box 77, Ghana; d.ankrah@kbth.gov.gh (D.A.); Waggor23@yahoo.com (A.A.); 8 Pharmacy, Northumbria Healthcare NHS Foundation Trust, North Shields NE29 8NH, UK; scott.barrett@northumbria-healthcare.nhs.uk (S.B.); joseph.brayson@northumbria-healthcare.nhs.uk (J.B.); 9 Pharmacy Department, Kilimanjaro Christian Medical Centre (KCMC), Moshi P.O. Box 3010, Tanzania; e.muro@kcri.ac.tz (E.M.); peterbenedict55@gmail.com (P.B.); 10 Pharmacy, Cambridge University Hospitals (CUH), NHS Foundation Trust, Cambridge CB2 0QQ, UK; reem.santos@nhs.net; 11 Pharmacy Department, Mulago Specialised Women and Neonatal Hospital Kawempe Hospital, Kampala P.O. Box 22081, Uganda; rosekantu61@gmail.com (R.K.); ronaldonegwa@yahoo.com (R.O.);; 12 Department of Obstetrics and Gynaecology, Makerere University and Mulago National Referral Hospital, Kampala P.O. Box 22081, Uganda; msekikubo@gmail.com; 13 Pharmacy, Brighton and Sussex University Hospitals NHS Trust (BSUH), Brighton, BN2 5BE, UK; Fiona.rees2@nhs.net; 14 Pharmacy Department, University Teaching Hospital (UTH), Lusaka P/Bag RW 1X, Zambia; banda.chimbi@gmail.com (D.B.); chichokalungia@gmail.com (A.C.K.); alutuliluke@yahoo.co.uk (L.A.); echiks@yahoo.com (E.C.); 15 Department of Pharmacy, University of Zambia, Lusaka P.O. Box 50110, Zambia; 16 Commonwealth Pharmacy Association (CPA), London E1W 1AW, UK

**Keywords:** antimicrobial stewardship, antimicrobial resistance, CwPAMS, AMR

## Abstract

The World Health Organisation (WHO) and others have identified, as a priority, the need to improve antimicrobial stewardship (AMS) interventions as part of the effort to tackle antimicrobial resistance (AMR). An international health partnership model, the Commonwealth Partnerships for Antimicrobial Stewardship (CwPAMS) programme, was established between selected countries in Africa (Ghana, Tanzania, Zambia and Uganda) and the UK to support AMS. This was funded by UK aid under the Fleming Fund and managed by the Commonwealth Pharmacists Association (CPA) and Tropical Health and Education Trust (THET). The primary aims were to develop local AMS teams and generate antimicrobial consumption surveillance data, quality improvement initiatives, infection prevention and control (IPC) and education/training to reduce AMR. Education and training were key components in achieving this, with pharmacists taking a lead role in developing and leading AMS interventions. Pharmacist-led interventions in Ghana improved access to national antimicrobial prescribing guidelines via the CwPAMS mobile app and improved compliance with policy from 18% to 70% initially for patients with pneumonia in one outpatient clinic. Capacity development on AMS and IPC were achieved in both Tanzania and Zambia, and a train-the-trainer model on the local production of alcohol hand rub in Uganda and Zambia. The model of pharmacy health partnerships has been identified as a model with great potential to be used in other low and middle income countries (LMICs) to support tackling AMR.

## 1. Introduction

Antimicrobial resistance (AMR) is a globally recognised threat to humanity and has been compared to a slow-burning version of the current COVID-19 epidemic. In 2019, it was recognised as one of the top 10 global health threats, and there is evidence that the COVID-19 pandemic is adding to the risk [1,2].

While substantial efforts have been made to combat AMR globally, concern has been raised in low- and middle-income countries (LMIC) due to the lack of resources, antimicrobial culture and sensitivity availability and increased utilisation of antimicrobials in these settings [3]. Data on AMR is scarce and incomplete in many LMICs due to a lack of funding, limited guidance and lack of surveillance. This makes it hard to quantify the issues and prioritise solutions.

In the UK, pharmacists play an important role in increasing the awareness of AMR and supporting the implementation of antimicrobial stewardship interventions and programmes (AMS) [4]. Pharmacists work alongside prescribers to ensure antimicrobials are prescribed when there is a clinical sign of infection and, when indicated, are prescribed appropriately. The pharmacist role in some LMICs is not as developed, with pharmacists less integrated into clinical multidisciplinary teams, resulting in their leadership potential often being overlooked [5]. Trained pharmacists in LMICs have the potential to play a role in leading antimicrobial stewardship programmes like their counterparts in the UK or USA for example and to be part of the solution to overcome the global challenge of AMR. Due to the training requirements, medical staff often have short periods of training before moving locations to continue their development. Pharmacists are often at posts for longer periods of time and so are ideally placed to lead long-term projects.

In 2019, the UK Fleming Fund funded and established the Commonwealth Partnerships for Antimicrobial Stewardship (CwPAMS), which uses a health partnership approach. This was managed by the Commonwealth Pharmacists Association (CPA) (as the technical leads) and Tropical Health and Education Trust (THET) (as grant managers) [6]. CwPAMS supported partnerships between health delivery institutions (including NHS bodies in the UK), academic institutions and other health institutions in Ghana, Tanzania, Uganda and Zambia to work together on antimicrobial stewardship (AMS) initiatives. The aim of the partnerships is to enhance the implementation of protocols and evidence-based decision-making to support antimicrobial prescribing and improve the capacity for antimicrobial surveillance to support tackling AMR. Specified within the call for partnership proposals by THET and CPA was a requirement to include pharmacists both in the UK and the four African countries to lead in developing and implementing antimicrobial stewardship interventions [7]. Pharmacists in the UK as part of the projects then linked directly with pharmacists in African countries to support their colleagues in Africa. Traditionally, volunteer opportunities in global health involve spending prolonged time in a country, and improvements are not always sustainable when the volunteer(s) leave [8]. As a novel approach with a strong focus on the pharmacy profession, pharmacists in the UK with limited funding and restricted time focused on building the capacity of pharmacists in Ghana, Uganda, Tanzania and Zambia in order to develop sustainable antimicrobial stewardship interventions.

Success through these projects has potential wider implications for antimicrobial stewardship in other LMIC. Five of the twelve funded commonwealth partnerships for antimicrobial stewardship are discussed in more detail in this paper, focusing on the impact of the local pharmacists taking a leadership role within these projects.

To further develop the global health leadership skills of pharmacists in the UK, Health Education England and CPA developed a new global health fellowship programme for pharmacists within the projects: The Chief Pharmaceutical Officers Global Health Fellowship [9]. This was set up to provide education on global health/the international development of global AMS, leadership skills and additional project management skills to enhance their own personal development.

## 2. Materials and Method

### 2.1. CwPAMS

CwPAMS is a health partnership programme funded by the UK aid Fleming Fund to support partnerships between the UK National Health Service and academic institutions and health institutions in LMICs to work together on tackling AMS initiatives globally.

The key eligibility criteria for selection or approval of these health partnerships were: the lead UK institutions must be formally recognised as a health education institution, regulatory organisation, NHS (if the UK) or public/not-for-profit (if overseas) hospital. This can include professional associations, and whilst an academic institution or professional association can act as the official lead for a grant, there must be clear joint leadership from an NHS hospital/institution.

The CPA as the technical lead assisted UK AMS pharmacists interested in the programme connect with hospital and academic pharmacists interested in implementing AMS programs in 4 different African countries through various national agencies (i.e., National Pharmaceutical Societies/National Antimicrobial Agencies) in Ghana, Tanzania, Uganda and Zambia to bid for a grant through CwPAMS. Of the twelve selected AMS partnerships that met the eligibility criteria and were awarded the AMS grants, the key aspects from five are presented in this paper. Multidisciplinary UK AMS teams travelled to Ghana, Tanzania, Uganda and Zambia to work in partnership with local health workers during the project period from February 2019 to April 2020. However, there were reciprocal visits from LMIC members to UK facilities with some partnerships to gain first-hand experience of practice in the UK.

Support from CPA included the production of a training videos to support the local production of hand sanitisers and the development of tools, e.g., an AMS checklist was developed for CwPAMS partnerships and a behaviour checklist developed in collaboration with the change exchange to identify the knowledge, attitudes and perspectives of healthcare staff related to antibiotic use and prescribing/administration. A new prescribing app called the CwPAMS app was also developed. The app provided, for the first time, easy access to national infection management resources, including Standard Treatment Guidelines (STG) for each country, to improve the appropriate prescribing in-line with the national and international guidelines. Additional resources made available through the app include the WHO essential medicines list, surveillance tools, AMS training and infection prevention and control (IPC) resources.

Each project group included at least one pharmacist from the UK and one local pharmacist working in partnership with multidisciplinary teams to support local teams. The remit of the pharmacists was to ensure education was delivered, antimicrobial usage surveillance was undertaken where possible and local working structures were in place to deliver antimicrobial stewardship training to the wider hospital staff and to support long-term antimicrobial stewardship. Multidisciplinary project groups agreed how long UK pharmacists would be present in the country, as this was not predetermined by the grant. The time of the UK pharmacists in the country varied from three five-day visits to one or two visits of longer durations, such as 3 weeks. A summary of the number of pharmacists in each project and the time the UK pharmacists spent in their partnership country is included in Table 1. The duration of visits was short, as such, the majority of communication was conducted though remote working technology/virutally. Projects reported were implemented over the period of a year, from February 2019 to April 2020. In addition to the education and training and surveillance that was undertaken, all projects also developed a local focus for improvement. The projects were selected based on the local priorities agreed on within the multidisciplinary teams and were used to engage staff in and improve AMS.

### 2.2. Ghana

#### 2.2.1. Quality Improvement Project Keta Municipal Hospital (KMH)

A hospital antibiotic stewardship group was established in KMH to monitor antibiotic use. A Quality Improvement project was initiated and ran from October 2019 to June 2020. The aim of the project was to achieve 70% compliance with the antibiotic policy in outpatient prescriptions for pneumonia by June 2020.

Compliance with the antibiotic policy was assessed for prescriptions issued to ambulatory patients presenting in the outpatients’ clinic with a diagnosis of mild or moderate pneumonia who received oral antibiotics. Patients admitted to hospital or who received IV antibiotics for pneumonia were excluded.

The outcome measured was the percentage of compliance with the outpatient antibiotic policy for pneumonia prescriptions. Compliance with the antibiotic policy was determined based on the choice of antibiotics, and the antibiotic dose and duration were not assessed. Microbiology results were not available for patients to assess if the empiric antibiotic treatment was appropriate.

The total number of outpatients prescribed antibiotics for pneumonia per week (Monday–Friday) were retrieved from the hospital’s e-prescribing software database. Twenty patients per week were then sampled and assessed using a checklist to record the antibiotics prescribed and compliance with policy. A systematic sampling method was employed in the weekly prescription selection to ensure that prescriptions written by all prescribers (one specialist doctor, four medical doctors and four physician assistants) who attended to patients within a week were part of the sampled prescriptions. A proportional sampling approach based on the number of prescribers within the week was used. The data collection was performed by a pharmacist who was trained to extract the data from the database and was entered onto an excel spreadsheet. Pharmacists assessed prescription compliance with the antibiotic policy.

Quality Improvement methodology is a recognised systematic and iterative approach to generate the maximum change in behaviour. Similar projects have also been shown to improve antimicrobial prescribing using these methods in respiratory patients [10]. The project followed Quality Improvement methodology using Plan Do Study Act (PDSA) cycles to test solutions on a small scale. The interventions tested during the project included improving the prescriber’s access to the antimicrobial guidelines by training them on the use of the CwPAMS app and adapting and agreeing to the local antibiotic policy with prescribers, which were then displayed widely for easy access via posters in all working areas. After an initial project meeting for prescribers that included training on the AMS principles, monthly feedback was given on their progress. In addition, barriers to change were discussed with prescribers, and the overall outpatient compliance was also routinely discussed with prescribers individually.

#### 2.2.2. KBTH 12 Pharmacists Were Trained as CwPAMS App Superusers

The NMUH (UK) team visited KBTH (Ghana) in June 2019 and introduced the CwPAMS app to the pharmacy department. Twelve pharmacists were trained on its use, and they were then asked to train and recruit their pharmacist and doctor colleagues and encourage them to download it onto their smart devices.

The 12 superusers were provided with a data collection tool and recommended script to use when promoting the app. This included advise that they would receive a follow-up email from the NMUH team.

With permission, all names and email contact details of newly recruited colleagues were forwarded to the NMUH lead pharmacist. Momentum was maintained with monthly emails and WhatsApp© messages to the 12 superuser pharmacists.

The 12 superuser colleagues further promoted the app to the entire hospital during the World Antimicrobial Awareness week activities in November 2019, using the posters and advertising material provided by CwPAMS. The CwPAMS app metrics were obtained from data collected by the Horizon Strategic Partners. They assessed the frequency of page hits, guide opens and the number of registered users and downloads. 

A 30-point questionnaire was developed based on previously published questionnaire [11] and distributed electronically via SmartSurvey^®^. Recruited KBTH colleagues were emailed the survey and asked to complete it over a four-week period between June and July 2020. The results of the survey were analysed using Microsoft Excel for Office 365©.

#### 2.2.3. Development of Antimicrobial Management Guidelines Ghana Police Hospital (GPH)

The Health Improvement Scotland (UK) visited GPH in May 2019 to partner with a multidisciplinary team of health professionals and facilitate the AMS program using the Scottish Triad Approach: Information, Quality Improvement and Education [12]. A multidisciplinary antimicrobial stewardship committee was established that developed an action plan for the further development of AMS in GPH. This included conducting a Point Prevalence Survey (PPS) of antimicrobial prescribing and development of the identified improvements and guidelines required [13]. The Global-PPS (GPPS) study was conducted for the first time in May 2019 for one day on all inpatients that stayed overnight and were present at 8:00 a.m on survey day. The data was collected manually from prescription charts and patient notes. The data was then entered on the GPPS web-based application. Antimicrobial utilisation was assessed against the quality indicators, including:

- indication of antimicrobial use documented in the patient notes;
- compliance with guidelines for documented indication (where guidance was available);
- missing/undocumented guidelines;
- documentation of stop or review date for antimicrobials in the notes (and prescription chart);
- proportion of surgical prophylaxis prescribed for less than or greater than 1 day.

The guidelines used were the seventh edition of the national STGs of Ghana.

### 2.3. Tanzania: Observership and Subsequent Pharmacy Leading Delivery of AMS at Kilimanjaro Christian Medical Centre (KCMC) and within Local Community

The core team consisted of pharmacists from Northumbria Healthcare NHS Trust (NHCT) and Kilimanjaro Christian Medical Centre (KCMC) supported by an MDT with IPC nurses, microbiologists and pathologists.

At the outset of the KCMC-NHCT partnership, a core team of pharmacists (as well as microbiologist and medical director from KCMC) undertook a 4-week observership at NHCT, including professional shadowing and a leadership development program providing them with the skills and confidence to take the lead in the following AMS project. The programme designed is available as Supplementary Materials 4 (KCMC Visit to UK_Skeleton programme 03.5.19).

The project adopted a multifaceted approach encompassing a wide range of interventions beginning with a robust point prevalence survey to provide antimicrobial usage data that highlighted the focus for education. Materials such as lectures, workshops and posters were developed and translated into Swahili for delivery. Dedicated workshops were organised for hospital staff, and the team added antibiotic awareness training for the first time as part of the school general education trips by hospital staff and traffic accident training for Boda boda drivers.

### 2.4. Uganda: AMS Training and Local Production of Alcohol-Based Hand Rub (ABHR)

The Kampala Cambridge Antimicrobial Stewardship (AMS) and Infection Prevention and Control (IPC) partnership brought together healthcare staff from various disciplines, including management, medicine, nursing and pharmacy. The project set out to reduce healthcare-associated infections in the obstetric and neonatal wards at the Kawempe National Referral Hospital (KNRH) and Mulago Specialised Women and Neonatal Hospital (MSWNH) through an interactive educational package. The content of the training was developed in collaboration with Ugandan partners and two behavioural scientists and focused on clinical and management training in antimicrobial stewardship and infection control. A unique core element of the training centred around promoting the value of pharmacists in multidisciplinary settings, with the aim of promoting future inter-collaborative working. This was achieved through individualised group activities and role play. The materials and equipment used in the training were shared with the Kampala partners to support them in developing local training with oversight from their medicines and therapeutics committee.

IPC is an integral part of global and national action plans to tackle AMR, and hand hygiene is a key component to reducing the spread of infection. Thus, enhancing the availability and the appropriate use of ABHR among health workers remains part of the key AMS strategies in healthcare facilities, especially in LMICs. With the support of the health partnership and hospital administrators, one pharmacist at KNRH received training at a regional district hospital in the large-scale manufacturing of alcohol-based hand rubs to meet the needs of their organisation. A train-the-trainer model was then employed.

### 2.5. Zambia: Infection Prevention Control and AMS training

Brighton-Lusaka Pharmacy Link (BLPL) conducted a three-day conference in Zambia for national-level stakeholders and institutions interfacing with the national AMR strategy, including an AMS train-the-trainer workshop for UTH pharmacists, doctors, nurses and allied healthcare professionals to increase the awareness of AMS and provide capacity-building tools. This included detailing the UTH AMR patterns, use of point prevalence surveillance (PPS) methodology, multidisciplinary team (MDT) approaches, enhancing appropriate prescribing, IPC (including a bare below elbow (BBE) dress code) and AMS rollout training methodologies, therefore encompassing the World Health Organisation AMR prevention strategies.

To foster intra-country collaboration, BLPL approached Ndola Teaching Hospital (NTH) (who had previously been IPC trained via a partnership with Guys & St Thomas) to provide initial expertise and training for IPC, which UTH used for rollout and future trainings targeting healthcare workers in Zambia. Context-appropriate posters were developed and alcohol hand rub production capacity and facilities enhanced across three other tertiary level public teaching hospitals in the country. Three-day on-the-job IPC trainings facilitated by UTH and NCTH staff were conducted at UTH, LCTH, KCTH and LMUTH, respectively. This training included WHO-recommended hand sanitisation techniques, including when appropriate to be completed (i.e., the “five moments of hand hygiene”) for optimum effectiveness in the prevention of infection plus techniques of producing alcohol-based hand rubs using WHO modified formulations and its subsequent packaging, storage and utilisation.

UTH pharmacists led the development of a national AMS training manual, currently undergoing accreditation by the Health Professions Council of Zambia—the national regulatory body for continuous professions development (CPD) of health workers in Zambia.

Clinical audit training was commissioned to ensure UTH AMS MDT develop their skills in monitoring and evaluation and ensure ongoing project sustainability and development.

## 3. Results

### 3.1. CwPAMS

The video on using the WHO formulation was viewed 847 times on YouTube. Following the launch of the CwPAMS app for the 12 institutions across the four countries, there were 530 downloads of the app and 2795 guide opens within 12 months. Ghana had more page hits (50.3%) than Uganda (31%), Tanzania (13%), Zambia (1.9%) and others (3.8%) within 12 months.

### 3.2. Summary of Key Activities and Outcomes across the Five Partnerships

An overall summary of the activities by the partnerships included in this study is included in Table 2. All partnerships conducted AMS activities and delivered education. An initial/baseline point prevalence survey (PPS) of antimicrobial use was conducted in the institutions of four out of five partnerships, and a postintervention PPS was undertaken by two partnerships (Healthcare Improvement Scotland Partnership in Ghana and Northumbria Healthcare NHS Foundation Trust in Tanzania). As a result of the partnership, all institutions now have a functioning formal organisational multidisciplinary structure of AMS (e.g., committee or group) that focuses on or takes responsibility for appropriate antimicrobial use.

For each partnership, a description of one or two unique completed and/or ongoing activities is provided, and an assessment of the outcomes given.

### 3.3. Ghana

#### 3.3.1. Quality Improvement Project Keta Municipal Hospital (KMH)

**Figure 1:**
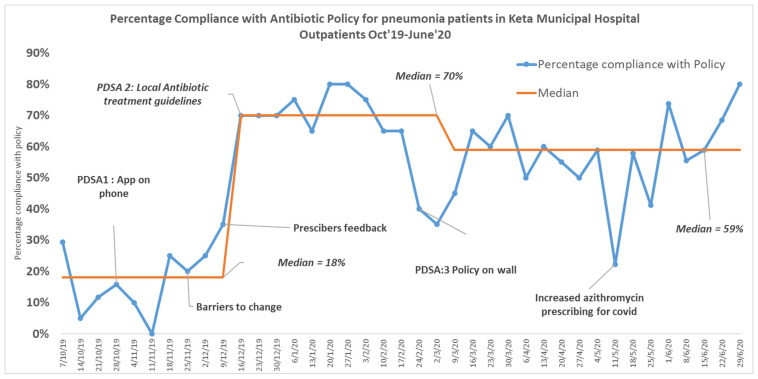
Percentage compliance with antimicrobial guidelines in outpatient prescriptions for pneumonia in Keta Municipal Hospital, Ghana.

A total of 757 prescriptions were assessed during the 39 weeks of the Quality Improvement project. Compliance with the antibiotic policy showed improvement from 18% to 70% within 3 months but then reduced to 57%, which was sustained until June 2020 (Figure 1). The aim of the project to achieve 70% compliance with the policy was met initially but not sustained. Improved compliance with the policy was not sustained due to a prescriber changeover just before the COVID-19 pandemic of approximately 60%. Planning for the COVID-19 response reduced the time available for the training of new staff. However, it was encouraging to see that compliance with the policy did not return to baseline at this time. Successful interventions were giving monthly feedback to prescribers both individually and in a meeting format and improving prescribers’ access to guideline through the display of antibiotic policy as posters on the wall in all working areas.

#### 3.3.2. Local Pharmacists Lead on Implementing the CwPAMS Antimicrobial Guidelines App at Korle-Bu Teaching Hospital (KBTH)

Fifty-five KBTH colleagues’ contact details were collected after they were recruited to download and use the app by twelve KBTH superuser pharmacists: 32 doctors, 16 pharmacists and 7 nurses. Of the 55 colleagues eligible to complete the survey, 16 were excluded due to incomplete contact details (five doctors, as they did not provide an email address, and 11 colleagues (10 doctors and one nurse) provided incomplete email addresses. Of the remaining 39 colleagues, less than half 17/39 (45%) responded to the survey. The majority (16) were pharmacists and one microbiologist: 16 heard about the app during the June 2019 visit, 11 used the app once a week and 4 had not used the app at all since downloading it. Thirteen stated that they found the app easy to navigate, and 14 reported that it was easy to recommend to colleagues.

**Figure 2:**
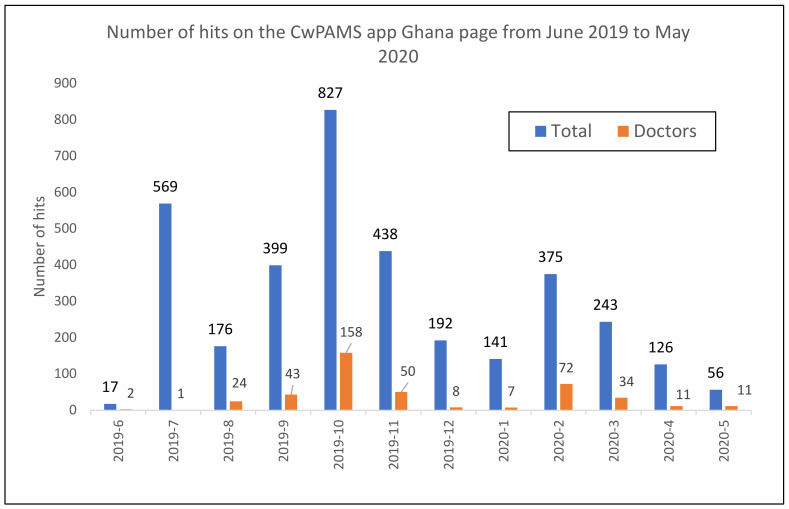
Number of hits of the Ghana Antimicrobial Guidelines page of the CwPAMS App.

Data from the app showed that the highest number of hits on the CwPAMS app Ghana page was in October 2019 (827), followed by July 2019 (569) and then November (438) 2019 (Figure 2). Hits by doctors were the highest in October 2019 (158), followed by February 2020 (72) and then November 2019 (50).

There was a low response rate to the survey to assess the effectiveness of the app from medical colleagues.

Issues with using the app were identified as technical issues with the app and lack of data/Wi-Fi. Although 16 respondents stated that they would recommend the app to colleagues, only three (17%) recommended the app to more than six people. The data for the app represented all hits on the CwPAMS app Ghana page, and Table 2 shows that other Ghana partnerships also promoted the app. The CwPAMS rollout of the app in Korle-Bu was in June 2019 and corresponded to the increase seen in July of people accessing the app. The education and promotion of the app in the other partnership hospitals during October corresponded to the October peak in use of the app (Figure 2). Lower levels of engagement with the app were seen starting in March 2020. This may be due to the pandemic and the fact that COVID19 guidance was not included, or it could have been due to prescribers already having the app.

#### 3.3.3. Development of Antimicrobial Management Guidelines Ghana Police Hospital (GPH)

The baseline PPS conducted at GPH in May 2019 as part of the CwPAMS project highlighted that, for many indications, guideline compliance could not be assessed due to the use of high levels of missing or undocumented guidelines (Table 3). This observation was prominent in two departments: obstetrics and gynaecology (OBGY) and surgery and formed the basis of the work plan developed (Supplementary Materials 1). Following further discussions with surgical and nursing teams, it was highlighted that the protocols for managing a range of conditions, including caesarean operations and post-delivery prophylaxis and surgical wound management, were in use but not available through the national standard treatment guidelines (STGs) or formally agreed-on local protocols.

To enable the assessment of appropriate prescribing, the AMS team led by the local pharmacist communicated the findings from the PPS to the hospital staff at a clinical meeting and highlighted the importance of having approved guidelines for caesarean, post-delivery prophylaxis and the management of surgical wounds in their respective facilities. To promote the acceptance and use of the guidelines in the unit, the AMS team collaborated with the various units through their personnel on the AMS to draft the final documents. The OBGY specialists and senior nurses developed the protocols for the OBGY, and the surgeon on the AMS team, who was also the head of the surgical department, led the development of the draft wound management protocols with the surgeons in his unit based on their experience/training (Supplementary Materials 2). For both guidelines, the local pharmacist as part of the AMS Committee in GPH worked with the senior medical/specialist and nursing staff to develop antimicrobial management guidelines within the protocols by assessing the appropriateness of dose, frequency and duration using the British National Formulary, as well as the protocols used at one of the Ghana Tertiary National Referral Hospitals (Korle-Bu Teaching Hospital). These guidelines were then implemented within the hospital. The antibiotics and other drugs required are now included on pre-printed patient medications lists issued routinely for patients receiving caesarean delivery, normal delivery and for those who require post-delivery prophylaxis (Supplementary Materials 3).

The adherence to the guidelines developed for the obstetrics and gynaecology department from the results obtained from the post-intervention PPS carried out in February 2020 was found to be 100%. The overall comparison of the compliance by prescribers to the national STG from PPS conducted at GPH in February (2020) revealed an increase from 64.6% in May (2019) to 88%.

### 3.4. Tanzania: Observership and Subsequent Pharmacy Leading Delivery of AMS at Kilimanjaro Christian Medical Centre (KCMC) and within Local Community

**Figure 3:**
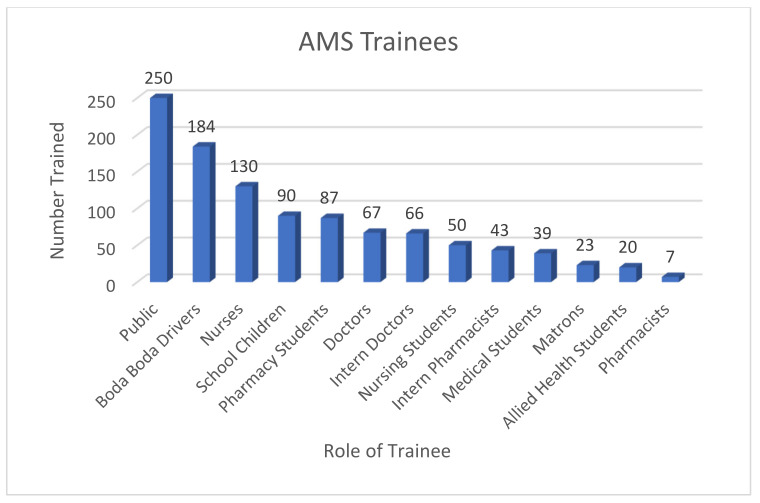
Range of trainee roles and numbers of trainees receiving AMS training in Tanzania.

AMS and IPC training was delivered to a total of 1056 people, including healthcare professionals at KCMC, and to the wider community, including schools and boda boda taxi drivers (Figure 3). More than one-third of participants in training sessions (36%) were not aware of the main principles of AMS before training. The education campaign then extended beyond the scope of the sessions above, with links being set up between the Kilimanjaro School of Pharmacy and Newcastle University Pharmacy School. These resulted in a joint AMS module being developed for delivery to early years pharmacy trainees, ensuring that pharmacists of the future continue to lead on AMS activity within Tanzania and the UK. This module is also being considered for adaptation to medical schools and dentistry schools within Moshi, Tanzania.

Furthermore, the educational lead pharmacist at KCMC, who took part in the four-week observership (Supplementary Material 4), was able to share the work done within the project and went on to be appointed the Chair of the National Tanzania AMS Council.

### 3.5. Uganda: Local Production of Alcohol Hand Rub

Forty-two healthcare workers received the initial training in AMS: five were pharmacists. Using the train-the-trainer model, the pharmacist with large-scale alcohol-based hand rub training then trained five pharmacist interns on how to produce the hand rub from March to September 2020, and a second cohort of interns (six in total) were undergoing training at the time of publication. After receiving the AMS and IPC training, hospital administrators and managers were found to be more receptive in supporting pharmacists with the local onsite production of alcohol hand rub initially at one hospital and then rolling out in a second hospital.

**Figure 4:**
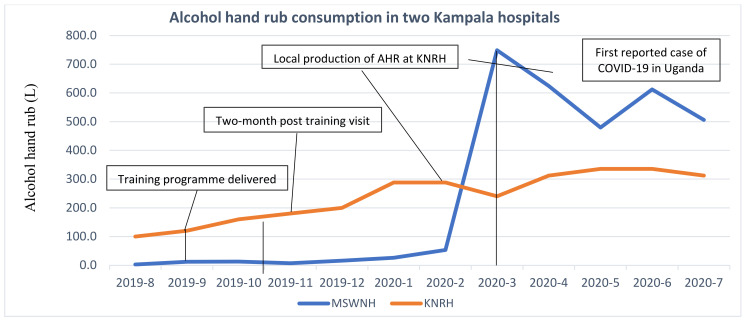
Alcohol consumption in 2 hospitals in Kampala, Uganda.

In the absence of monthly audits of hand hygiene compliance, a crude measure of alcohol hand rub purchases was used as a proxy for consumption to measure the success of the interventions. Where pure ethanol (96%) was procured by Kawempe National Referral Hospital (KNRH) for alcohol hand rub manufacture, the equivalent final volume was used based on the WHO formula (reference below). Purchases of alcohol hand rub by pharmacists also increased at the second partner hospital Mulago Specialised Women and Neonatal Hospital (MSWNH) as part of a multifaceted approach to improve IPC among healthcare workers and staff (see Figure 4).

### 3.6. Zambia: Infection Prevention Control and AMS Training

In total, 297 healthcare workers from a variety of professions (including pharmacists, doctors, nurses, allied health workers, hospital administrators, porters and cleaning staff) were trained in IPC, with 36 more trained in hand rub production at the four participating hospitals, respectively (Table 4).

The project foundations have coped with demand, with alcohol hand rub production increasing to over 120 litres per day. A local “bare below the elbow (BBE)” dress code for health professionals on the wards was implemented, and BBE practice was also endorsed nationally by the Hospital Pharmacists Association of Zambia (HOPAZ).

Additional equipment was procured and installed at the four tertiary hospitals to develop alcohol hand rub production facilities (using a WHO-approved formula) and alcohol hand rub dispensers installed in UTH wards.

With pharmacists leading, an AMS training manual was developed by the CwPAMS project team to facilitate further MDT education and continuous professional development (CPD) in AMS among healthcare workers. The AMS training modules were validated by local stakeholders and are currently being reviewed for University of Zambia (UNZA) accreditation.

## 4. Discussion

Empowering pharmacists in Ghana, Uganda, Tanzania and Zambia to lead AMS activities and rise in awareness of AMR through structured collaboration with UK pharmacists have had a positive impact. Pharmacists within the participating countries have raised the awareness of AMR as well as developed and delivered several AMS interventions, including education and training and conducting PPS. They have locally led AMS interventions through engaging with colleagues, e.g., the cascade of the CwPAMS app, an Antibiotic Guardian pledge-based campaign, WAAW activities and guideline compliance, and they have also engaged with the public, e.g., Boda boda drivers in Tanzania. Using a quality improvement approach, helped in the identification of prescribing barriers, such as low trust in generic medication, fear of treatment failure with first-line antibiotics and lack of awareness of policy. Sociocultural barriers of accessing antibiotic policy using the CwPAMS Antimicrobial Guideline app through mobile phones while taking the patient history for fear of being seen to be using social media were also identified, allowing the pharmacists to work with their prescriber colleagues to address the local barriers to change. Engaging staff over a longer period of time with regular feedback, similar to other studies, incorporating the feedback of surveillance data collected from, e.g., PPS within projects, was shown to lead to positive effects, including the development of new protocols led by clinicians with support from the pharmacists as part of institutions’ AMS teams [12,13]. In addition, pharmacists also led IPC interventions, including Bare Below the Elbow (BBE) campaigns and the large-scale production of alcohol hand rub during the COVID-19 pandemic [14]. Bidirectional learning was also evident particularly during COVID-19 management in the UK, where pharmacists in Zambia trained UK pharmacists to locally produce alcohol hand rub using the WHO hand rub formula. The pharmacists that were part of the CwPAMS projects are now considered core members of their respective AMS committees, providing a sustainable model for AMS within these hospitals. Using a variety of methods and approaches but always focusing on local priorities and being led by local pharmacists will have an impact on patient care, improving infection management and supporting the actions to tackle AMR. Like their colleagues in the UK [4], they now play an important role in leading AMS within their institutions.

The collaboration not only highlighted the importance of tackling AMR, but it also raised the profiles of the pharmacists, resulting in their unique skills as pharmacists being recognised within the MDT environment. The skills and experiences gained during the project have given local pharmacists the opportunity to expand their roles. Some pharmacists have taken on a more clinical pharmacy role. Many are now embedded as key members of MDT clinical teams, working with their prescribing colleagues to improve compliance with the antibiotic policy and conduct ward rounds. Pharmacists involved in the projects were also able to link with national committees to support national efforts to tackle AMR through AMS and the surveillance of antimicrobial use, as well as working with external organisations, including universities, to develop modules for pre- and post-service education on AMR. An example is in Tanzania, where the education lead pharmacist and member of the CwPAMS project at KCMC was recently appointed Chair of the National Tanzania AMS team and has successfully introduced AMS training modules as part of the healthcare school’s curriculum within medicine, pharmacy and dentistry.

The approach of collaborating via different methods with a focus on education and training and the rollout of a train-the-trainer approach reduced the overall cost and the time pharmacists from the UK required to be in partnership countries. This was a successful approach that could be replicated elsewhere to improve AMS. Education resources from the CwPAMS project and CPA will make it easier for others to be able to increase the awareness of AMS in other LMIC and replicate the local engagements, finding their own focus for improvements in AMS.

While there have been many successes in the projects, these have not been without challenges. In Zambia, funding was a barrier initially to scale up the alcohol hand rub production to other facilities. Barriers with prescriber attitudes, the use of apps due to access to internet data and space app took up on the phones and communication within the multidisciplinary team (MDT) were also identified as issues within the Ghana project. Traditional pharmacy roles need to be challenged in order to allow pharmacists to fulfil their potential within the MDT environment. Ongoing support will be needed to ensure sustainability and continued support from hospital managers. Government support will also be required to sustain the IPC interventions, particularly with the current COVID-19 pandemic.

These projects have been very successful at the local level, but AMR awareness and the benefits of AMS are still not widely understood or discussed, and more needs to be done to join up those working locally to teams working within governments to tackle AMR nationally but, also, join up with the effort internationally in order to engage more people in AMR and AMS conversations. Urgency is needed to get the message heard on a larger scale. WHO has recognised AMR as a global health threat, and the barriers need to be overcome in order to respond with a global response to this issue. CPA have made great strides in this aim by both supporting this work and by providing practical advice and resources for pharmacists in LMIC, including the CwPAMS toolkit and checklist (https://commonwealthpharmacy.org/cwpams-toolkit/, accessed on 1 November 2020). The newly released CPA continuing the professional development platform, which has an antimicrobial module supporting pharmacists in their efforts to reduce AMR by providing educational and networking opportunities for pharmacists. The CwPAMS toolkit brings together resources and shared experiences from the CwPAMS projects within the framework of the Core Elements for AMS. It also aims to signpost published works or provide CwPAMS project resources to support each component of the Core Elements of AMS. The toolkit may be used by healthcare facilities to identify their own AMS priorities and implement a workplan on the local level. While this is a step in the right direction, more is still needed to spread the AMR message further.

One of the strengths of this study is that it adds to the body of evidence on the implementation of AMS interventions/programmes, especially within African countries [15,16,17,18,19,20,21,22,23]. A recent systematic review that focused on the extent of the implementation of stewardship programmes in Africa identified only thirteen primary studies: five studies from three countries in East Africa (Tanzania, Kenya and Sudan) and one study from Egypt in North Africa, with no studies identified within West Africa, although the region has the greatest number of countries in Africa [15,16,17,18,19,20,21,22,23].

The limitation of some of the data presented is that it includes, in some examples, small sample sizes and preliminary data, due to the fact that these are examples of the pharmacy health partnership model. This makes it difficult to draw conclusions from the data presented; however, it provides an illustration of the progress made within the partnerships. The COVID-19 pandemic had substantial impact on most projects, as it has with much improvement work throughout the world at this time. In particular, the rollout and spread of initiatives and data collection were impacted. The need to focus on COVID- 19 pandemic response has had a negative impact on implementing AMS interventions in a similar manner to many countries globally [24]. This also prevented the collection of qualitative feedback that would have improved the evidence available for the impact of the projects.

The model of UK pharmacists supporting colleagues within African countries has been identified as a model with great potential to be used widely both with other LMICs trying to tackle AMR but, also, for the further development in other areas of pharmacy where UK pharmacists are well-established, e.g., clinical pharmacy roles.

## 5. Conclusions

Empowering pharmacists in Ghana, Tanzania, Uganda and Zambia to lead within their own healthcare settings supported by their pharmacist colleagues in the UK encouraged them to embrace their antimicrobial stewardship role and enhanced the clinical pharmacy practices within the organisations and raised the pharmacy profiles both locally and nationally. Every country and hospital had a different focus for improvement based on local needs, from improving prescription developing guidelines and establishing new roles for pharmacists to a renewed focus on IPC and novel approaches to resolving local problems with local solutions. All local pharmacists in the projects embraced their new roles of promoting antimicrobial stewardship and ensuring the improvements made in partnership with their colleagues in the UK are sustainable. This shows that the model used is a model that has great potential for further development in the future. The examples shared within this article have the potential to enable the setup of similar, pharmacist-led initiatives in other LMIC institutions.

## Figures and Tables

**Table 1:** Summary of the projects undertaken.

Section	Country	Location	Local Project(s)	Number of Pharmacists Involved in Project (Includes UK and Partnership Pharmacists)	Time of UK Pharmacists in Country
2.2.1	Ghana	Keta Municipal Hospital (KMH)	Quality improvement project to improve compliance with antibioitic policy in pneumonia	9 (6 UK pharmacists; across KMH and GPH)	15 days in divided trips. 1st visit 5 days 1 pharmacist, 2nd visit 6 pharmacists 5 days and 3rd visit 3 pharmacists 5 days.
2.2.2		Korle- bu Teaching Hospital	CwPAMs Superusers	3 (1 UK pharmacist)	7 days
2.2.3		Ghana Police Hospital (GPH)	Development of antimicrobial Guidelines	9 (6 UK pharmacists; across KMH and GPH)	15 days in divided trips 1st visit 5 days 1 pharmacist, 2nd visit 6 pharmacists 5 days and 3rd visit 3 pharmacists 5 days.
2.3	Tanzania	Kilimanjaro Christian Medical Centre (KCMC)	Observership	6 (4 UK Pharmacists)	14 days days each
2.4	Uganda	Kawempe National Referral Hospital (KNRH) and Mulago Specialised Women and Neonatal Hospital (MSWNH)	Infection Prevention Control and AMS training	5 (3 UK pharmacists and 1 UK pharmacy technician)	21 days each for pharmacists and 7 days for pharmacy technician
2.5	Zambia	University Teaching Hospital (UTH), Lusaka	AMS training and local production of alcohol based hand rub (ABHR)	13 (3 UK pharmacists)	3 to 5 days each

**Table 2:** Summary of the activities by each partnership (✅ represents activities that took place).

Country	Ghana	Ghana	Tanzania	Uganda	Zambia
Partnership institutions	Healthcare Improvement Scotland–Ghana Police Hospital and Keta Municipal Hospital	North Middlesex University Hospital NHS Trust, London (NMUH)–Korle-Bu Teaching Hospital (KBTH) Healthcare Improvement	Northumbria Healthcare NHS Foundation Trust–Kilimanjaro Christian Medical Centre (KCMC)	Makerere University, Mulago Specialised Women and Neonatal Hospital, Kawempe Hospital and Cambridge	Brighton and Sussex University Hospitals NHS Trust (BSUH), Brighton and Sussex Medical School (BSMS) Brighton–University Teaching Hospital (UTH), University of Zambia (UNZA) Department of Pharmacy, Lusaka and University Hospital Ndola
AMS activity/intervention					
Baseline Point Prevalence Survey (PPS) completed	✅	✅	✅		✅
Post intervention PPS completed	✅		✅		
Assessment of institutions’ AMS activities using the AMS Checklist	✅	✅	✅	✅	✅
CwPAMS app promoted/implemented in institution	✅	✅	✅	✅	
Introduced principles of World Health Organisation AWaRe categories for antibiotics	✅	✅	✅	✅	
Antibiotic Guardian pledge-based campaign actively promoted	✅	✅	✅	✅	
Activities for World Antibiotic Awareness Week (WAAW) 2019	✅	✅	✅		
Activities for WAAW 2020	✅	✅			
Education and training					
Education on AMS principles	✅	✅	✅	✅	In development
Education on IPC		✅	✅	✅	✅
Education module Developed			✅		✅
Behaviour Change					
Health Psychologist input into developing AMS interventions	✅			✅	
Used Behaviour change methodology in developing AMS interventions	✅			✅	✅
Other Outcomes					
Committee established or re-energised responsible for AMS	✅	✅	✅	✅	✅
Infection management/AMS guidelines developed	✅				✅
Quality Improvement methodology	✅				
Audit training					✅
Antimicrobial Chart development					✅

**Table 3:** Quality indicators for AMS assessed as part of the PPS in May 2019 in Ghana Police Hospital.

	% Patients	
Quality Indicator	Medical (*n* = 31)	Surgical (*n* = 31)
Indication for Antibiotic Use Recorded	100	85
Guidelines missing	46	70
Guidelines Compliant	63	67
Stop/review date documented in notes	93	95
Surgical prophylaxis > 1 day	-	69

Key: Total patients (*n*) on antibiotics from the medical (31) and surgical (31) units = 62.

**Table 4:** Training in alcohol hand rub production and ICP.

Hospital	Number and Role of Those Trained in Alcohol Hand Rub Production	Quantity of Alcohol Hand Rub Produced after Initial Training	Number and Role of Those Trained in IPC
University Teaching Hospitals (UTH)	8 Pharmacists	100 litres	85 Nurses 14 Support staff 8 Pharmacists 4 Medical Doctors 2 Environmental Health Psychologist Technologist
Livingstone Central Hospital (LCH)	4 Pharmacists 3 Pharm. Tech. 1 Lab. Tech. 1 Env. Tech.	60 litres	36 Nurses 16 Pharmacists 3 Pharmacy Technologist 3 Support Staff 2 Physiotherapists 2 Administrative Officers 1 Medical Doctor
Kitwe Central Hospital (KCH)	9 Pharmacists	140 litres	24 Nurses 12 Pharmacists 11 Support staff 7 Medical Doctors 5 Environmental Health Psychologist Technologist 1 Laboratory Scientist 1 Pharmacy Technologist
Levy Mwanawasa University Teaching Hospital (LMUTH)	10 Pharmacists	130 litres	28 Nurses 14 Pharmacists 8 Lab Scientists 7 Physiotherapists 2 Medical Doctors 1 Support Staff
TOTAL	36	430 litres	297

## Data Availability

Data supporting reported results are available on request from the authors.

## Supplementary Materials

The following are available online at https://www.mdpi.com/article/10.3390/pharmacy9030124/s1. Supplementary material 1: Workplan based on the baseline GLOBAL-PPS study results at Ghana Police Hospital; Supplementary material 2: Antimicrobial Therapy in Wounds; Supplementary material 3: OBGY Drug List; Supplementary material 4: KCMC Visit to UK Skeleton programme 03.5.19.

## Abbreviations

WHO	World Health Organisation
CwPAMS	Commonwealth Partnership for Antimicrobial Stewardship
THET	Tropical Health Education Trust
CPA	Commonwealth Pharmacists Association
AMR	Antimicrobial Resistance
AMS	Antimicrobial stewardship
LMIC	Low- and middle-income countries
STG	Standard Treatment Guidelines
IPC	Infection prevention and control
PPS	Point Prevalence Survey
AWaRe	Access, Watch, Reserve
WAAW	World Antibiotic Awareness week
PDSA	Plan, Do Study, Act
KBTH	Korle-Bu Teaching Hospital
GPH	Ghana Police Hospital
KMH	Keta Municipal Hospital
OBGY	Obstetrics and Gynaecology
KCMC	Kilimanjaro Christian Medical Centre
MDT	Multi-disciplinary team
KNRH	Kawempe National Referral Hospital
MSWNH	Mulago Specialised Women and Neonatal Hospital
UTH	University Teaching Hospital
NCTH	Northumberland Healthcare NHS Trust
NTH	Ndola Teaching Hospital (NTH)
BBE	Bare below elbow
HOPAZ	Hospital Pharmacists Association Zambia
LCH	Livingston Central Hospital
KCH	Kitwe Central Hospital
LMUTH	Levy Mwanawasa University Teaching Hospital
UNZA	University of Zambia
CPD	Continued Professional Development
MTC	Medicines and Therapeutics Committee
BSUH	Brighton and Sussex Medical School
BLPL	Brighton–Luska Pharmacy Link
