# Supplementary material for: Practical Pharmacist-Led Interventions to Improve Antimicrobial Stewardship in Ghana, Tanzania, Uganda and Zambia
Source: Pharmacy (Basel). 2021 Jul 8;9(3):124. doi: 10.3390/pharmacy9030124 (PMC8293468; doi:10.3390/pharmacy9030124)
Supplement: Supplementary file 1 [file pharmacy-09-00124-s001.zip › Supplementary material 1_Workplan based on the baseline GLOBAL-PPS study results at Ghana Police Hospital.pdf]

Workplan based on the baseline GLOBAL-PPS study results Case Study at Ghana Police Hospital

Stewardship Quality Indicator	Objective	Current Position	Actions	Timeframe	Comments
Indication for antibiotic use recorded	To achieve > 95% compliance with documentation	Medical wards achieved<br><br>To increase to >95% on surgical wards	• Feedback to clinical teams<br>• Investigate reason for variation<br>• Identify missing /undocumented guidelines, develop and implement surgical (including) prophylaxis guidelines<br>• Re-measure	3-6 months	Variation may be due to extended surgical antibiotic prophylaxis, noting that surgical prophylaxis guidelines were missing in 70% of cases audited
Antibiotic treatment and prophylaxis should follow guidelines unless there is a strong clinical reason	To achieve >75% compliance with guidelines at the next audit	Medical wards 62.5%<br><br>Surgical wards 66.7%<br><br>High proportion of guidelines not available (70% on surgical ward)	Short-term Priority<br>• Implement guidance on timing and duration as per WHO global guide<br>• Investigate why guidelines are not followed e.g. stock availability, awareness of guidelines, access to guidelines<br>• Feedback results<br>Long term Priority<br>• Develop surgical prophylaxis guidelines based on WHO global guide where not available	6 months<br><br>1-2 years	The reasons for not following the guidelines will determine the AMS actions for example:<br>Lack of awareness / access<br>Actions:<br>• Promote CwPAMS App<br>• Printed posters of most common infections available on wards<br>• Low adherence to NSTGs observed often with House officers (Interns) suggesting need to train/orientate them on AMS in

					hospital<br>• Education / awareness session<br>Can guideline development be done in partnership with any other institution to share resources
