# Supplementary material for: Practical Pharmacist-Led Interventions to Improve Antimicrobial Stewardship in Ghana, Tanzania, Uganda and Zambia
Source: Pharmacy (Basel). 2021 Jul 8;9(3):124. doi: 10.3390/pharmacy9030124 (PMC8293468; doi:10.3390/pharmacy9030124)
Supplement: Supplementary file 1 [file pharmacy-09-00124-s001.zip › Supplementary material 2_AntimicrobialTherapyinWounds.pdf]

GUIDELINE FOR ANTIMICROBIAL THERAPY IN WOUNDS

1. Clean wound

(i) Surgically incised wound under aseptic condition without pre-existing infection or opening into any hollow viscus.

Treatment: No Antibiotics Needed

2. Clean Contaminated Wound

(i) Surgically incised wound under aseptic conditions into a hollow viscus without spillage.

(ii) Wounds of any other kind by a clean implement/instrument/tool seen in less than 6 hours without obvious contamination by foreign material.

Treatment: 1. Inj Amosiclav 1.2g start

OR

2.Inj. Ceftriazone 1g Start

3. Contaminated Wound

(i) Surgically incised wound under aseptic condition into a hollow viscus with spillage of contents.

(ii) Any other wound of any kind with obvious contamination by foreign/external material.

(iii) Clean contaminated wound expose for more than 6 hoúrs.

Treatment: (i) IV Amoxiclav 1.2g 8hourly X 72hrs

+

(ii) IV Flagyl 500mg 8hourly X 72hrs

+

(iii) IV *Gentamicin 80mg 8 hours X 72 hours

OR

(iv) IV Ceftriazone 1g daily X 72 hours

+

(v) IV Flagyl 500mg 8hours X 72 hours

OR

(vi) IV Clindamycin 300MG 8 hours X 72 hours

+

(vii) IV *Gentamycin 80 mg 8 hours X 72 hours

Oral

(i) Tab Amosiklav 62 mg 12 hours X 5days

+

Tab Flagyl 500mg 8 hours X 5days

OR

(ii) Cap Clindamycin 300mg 8 hourly X 5days

+

Tab Ciprofloxacin 500mg 12 hourly X 5days

4. Dirty Wound

(i) Surgically incised wound with obvious pus collection or faecal contamination.

(ii) Other wounds with pus or abscess formation or cross contamination with external material e.g. soil.

(iii) Wet gangrene

Treatment: Admit Patient

(i) IV Amosiklav 1.2g 8 hourly X 72 hours

+

(ii) IV *Gentamicin 80mg 8 hourly X 72 hours

+

(iii) IV Flagyl 500mg 8 hourly X 72 hours

OR

IV Ceftriazone 2g daily X 72 hours

+

IV Flagyl 500mg 8 hourly X 72 hours

OR

IV Clindamycin 300mg 8 hourly X 72 hours

+

IV Ceftriazone 2g daily X 72 hours

(*Treatment beyond 72 hours should be tailored to culture and sensitive result if available after 72 hours*)

* *Ensure normal urine output/or normal BUE&Cr before commencing use.*
