# Supplementary material for: Practical Pharmacist-Led Interventions to Improve Antimicrobial Stewardship in Ghana, Tanzania, Uganda and Zambia
Source: Pharmacy (Basel). 2021 Jul 8;9(3):124. doi: 10.3390/pharmacy9030124 (PMC8293468; doi:10.3390/pharmacy9030124)
Supplement: Supplementary file 1 [file pharmacy-09-00124-s001.zip › Supplementary material 3_OBGY DrugList.pdf]

B

GHANA POLICE HOSPITAL

DEPARTMENT OF OBSTETRICS & GYNAECOLOGY

CAESAREAN SECTION (C/S) DRUG LIST   DATE & TIME....................................

NAME OF PATIENT...   AGE..................

SERVICE NO..............................   RANK..................................   RELATIONSHIP.................................

1.	RINGERS LACTATE IVF	2000ml	
2.	NORMAL SALINE IVF	1500ml	
3.	DEXTROSE SALINE IVF	1500ml	
4.	AMOKSICLAV IV	1.2g BD X 24HRS	
5.	METRONIDAZOLE IV	500mg TDS X 24HRS	
6.	PARACETAMOL IV	1g TDS X 24HRS	
7.	OXYTOCIN IV/IM	40 UNITS	
8.	MISOPROSTOL (CYTOTEC)	1000mcg	
9.	DICLOFENAC SUPPOSITORY	100mg BD X 5DAYS	
10.	VITAMIN K INJECTION	1mg STAT	
11.	AMOKSICLAV TABS	625mg BD X 5DAYS	
12.	METRONIDAZOLE TABS	400mg TDS X 5DAYS	
13.	CETIRIZINE TABS	10mg OD X5DAYS	
14.	DISPOSABLE GLOVES	1 BOX	
15.	URETHRAL CATHETER SIZE 16	1	
16.	VIOPLEX- T SPRAY POWDER	1	
17.	ONETOUCH SELECT GLUCOMETER STRIPS	1 PACK	
18.			
19.			
