# Supplementary material for: Practical Pharmacist-Led Interventions to Improve Antimicrobial Stewardship in Ghana, Tanzania, Uganda and Zambia
Source: Pharmacy (Basel). 2021 Jul 8;9(3):124. doi: 10.3390/pharmacy9030124 (PMC8293468; doi:10.3390/pharmacy9030124)
Supplement: Supplementary file 1 [file pharmacy-09-00124-s001.zip › Supplementary material 4_KCMC Visit to UK_Skeleton programme 03.5.19.pdf]

Draft programme: CwPAMS Project: Kilimanjaro Christian Medical Centre (KCMC) team visit to Northumbria Healthcare NHS Trust (NHCT)* 03/05/2019

Friday 3rd May	Saturday 4th May	Sunday 5th May	Monday 6th May	Tuesday 7th May	Wednesday 8th May	Thursday 9th May
KCMC team depart from JRO airport<br>Flight: QR1354	Flight number: BA 1328<br><br>16:00<br>Expected arrival into Newcastle airport from London Heathrow<br>Collected by Robert Hope and Brenda Longstaff<br><br>Transport to WGH accommodation<br><br>Food shop	11:00<br>UK pharmacist to show team around Newcastle	Bank holiday Monday<br><br>Rest Day:<br>Suggested activity<br>Explore Woodhorn Museum<br>Entry fee £7.00	7:45am collected by BL for transport to Cobalt<br><br>Trust induction<br>8:15 – 12:00<br><br>12:00-12:30<br>Lunch<br><br>12:30 – 15:00<br>Trust induction<br><br>15:30-17:00<br>KCMC Microbiologist<br><br>Bone infection audit<br>Ward 11<br>WGH<br><br>15:30 -17:00<br>Basic sign language class<br>WGH	KCMC Microbiologist<br>9:00-13:00<br>(Ward 3)<br>NSECH<br>KCMC Microbiologist<br>13:05- 13:30<br>Transport to NTGH<br><br>09:00-09:30<br>KCMC Pharmacists<br>CDiff MDT<br>(Education Centre NTGH Room 10)<br><br>10:00-12:00<br>KCMC Pharmacists<br>NTGH Pharmacy Department Tour –<br><br>13:00-14:00<br>KCMC Pharmacists<br>Tour of laboratory NTGH –<br><br>14:00 – 16:00<br>Microbiology speciality meeting<br>(Pathology seminar room) NTGH	8:30 Collected by BL for<br>transport to NTGH<br><br>10:00-11:00<br>All KCMC colleagues to join IPC meeting<br>NTGH IPC office<br><br>11:00 -13:00<br>Shadow IPC nurse<br>NTGH<br><br>14:00-16:00<br>Antibiotic steering group<br>(Seminar room, next to endoscopy)<br>NTGH<br><br>16:10<br>Return to WGH accommodation collection by RH<br><br>e

Draft programme: CwPAMS Project: Kilimanjaro Christian Medical Centre (KCMC) team visit to Northumbria Healthcare NHS Trust (NHCT)* 03/05/2019

Friday 10th May	Saturday 11th May	Sunday 12th May	Monday 13th May	Tuesday 14th May	Wednesday 15th May	Thursday 16th May
9:30 – 11:30<br>KCMC Microbiologist<br>KCMC Pharmacists<br>Bone infection ward rounds<br>WGH<br><br>11:00-12:00<br>KCMC Pharmacists<br>Lab sensitivity<br>NTGH<br><br>Collection of daily living allowance<br>Cashiers office<br>NTGH<br><br>13:00 – 17:00<br>KCMC Microbiologist<br>KCMC Pharmacists<br>AMR Project Planning<br>Room 11, Education Centre WGH	TBC	Visiting Medical Director<br>Visiting Microbiologist<br>Visiting Pharmacists<br><br>Informal activities and tourist activities with UK nurse Volunteer	8:30-12:30<br>KCMC Microbiologist<br>(Ward 3)<br>NSECH<br><br>8:30-12:30<br>KCMC Pharmacists<br>NSECH main entrance<br>Shadow Clinical Pharmacy Service<br><br>14:00- 16:00<br>KCMC Microbiologist<br>KCMC Pharmacists<br>Sepsis steering group<br>(Conference centre)<br>NSECH	Collected by RH<br>9:30 – 11:30<br>KCMC Microbiologist<br>KCMC Pharmacists<br>Bone infection ward rounds<br>WGH<br><br>14:00 -14:30<br>KCMC Pharmacists<br>HCI meeting<br>(Education room)<br>WGH Room 11<br><br>15:00 17:00<br>KCMC Pharmacists<br>With NTGH Senior Clinical Pharmacists<br>WGH pharmacy Clinical Service<br><br>KCMC Microbiologist<br>14:00-16:00<br>Acute medicine consultant meeting<br>(Ward 7 ED seminar room) NSECH	Collected by RH<br><br>KCMC Microbiologist<br>KCMC Pharmacists<br><br>9:00- 9:30<br>C Diff MDT<br>(Education centre)<br>NTGH<br><br>9:30-17:00<br>Day case unit<br>- Complications<br>- Infections<br>WGH<br><br>13:00 – 14:30<br>Clinical strategy group<br>(Conference centre)<br>Cobalt<br><br>14:00- 16:00<br>Emergency Medicine Operation Board meeting<br>(Seminar room)<br>NSECH	KCMC Microbiologist<br>KCMC Pharmacists<br><br>10:00-11:00<br>Meeting with NTGH Chief Pharmacist<br>(Pharmacy)<br>NTGH<br><br>13:00 – 16:00<br>Basic Drug awareness<br>(Room 6&7)<br>WGH

Draft programme: CwPAMS Project: Kilimanjaro Christian Medical Centre (KCMC) team visit to Northumbria Healthcare NHS Trust (NHCT)* 03/05/2019

Friday 17rd May	Saturday 18th May	Sunday 19th May	Monday 20th May	Tuesday 21st May	Wednesday 22nd May	Thursday 23rd May
KCMC Microbiologist<br>KCMC Pharmacists<br>Pharmacy University Student Training – (WGH Education Centre)<br><br>11:00 -12:00<br>Peter Benedict<br>Lab sensitivity NTGH<br><br>Collection of per diem daily living allowance<br><br>12:00-17:00<br>Northumbria Flow Celebration Day Holiday Inn Jesmond	Time :TBC<br>Afternoon tea at Alnwick Garden treehouse	15:00<br>Team meal at Ridley Arms	9:00-10:10<br>KCMC Medical Director<br>Sign business case, annex 4 and MOU<br><br>10:30-11:00<br>KCMC Medical Director Meeting with **Director of Nursing**<br>(Comms meeting room)<br><br>11:00-11:30<br>KCMC Medical Director<br>Meeting with NTGH Comms Officer<br>(Comms meeting room)<br><br>9:00-17:00<br>KCMC Microbiologist<br>Ambulatory care NSECH<br><br>9:00-17:00<br>KCMC Pharmacists<br>NSECH PPS Data Collection	8:30-12:30<br>KCMC Microbiologist with Infectious Disease clinic<br><br>13:30-14:30<br>KCMC Medical Director and KCMC Microbiologist<br>Lab tour NTGH<br><br>KCMC Pharmacists<br>With NTGH Pharmacy Clinical Service<br>8:30 – 13:00<br><br>14:00 – 14:30<br>HCI meeting<br><br>NTGH<br>14:30- 17:00<br>Medicine management committee<br><br>KCMC Medical Director Travel to London	9:00- 9:30<br>KCMC Microbiologist<br>KCMC Pharmacists<br>C Diff MDT<br>(Education centre, NSECH - video conferenced)<br><br>Report to Infection Prevention Control<br>09:30 – 12:00<br>Shadow IPC nurse NSECH<br><br>14:00 – 17:00<br>Regional antimicrobial pharmacist Group<br><br>Visiting Medical Director<br>THET Innovation steering group Royal society medicine	9:00 12:00<br>KCMC Pharmacists<br>Gosport Training & Findings. NTGH Pharmacy<br><br>KCMC Medical Director Return from London<br>12:30 arrival<br><br>KCMC Microbiologist<br><br>9:00-17:00<br>Shadow IPC nurse WGH<br><br>14:30-16:00<br>KCMC Pharmacists<br>Meeting with Pharmacy Department Newcastle University

Draft programme: CwPAMS Project: Kilimanjaro Christian Medical Centre (KCMC) team visit to Northumbria Healthcare NHS Trust (NHCT)* 03/05/2019

				16:35 departure		
Friday 24th May<br><br>10:00-11:00<br>KCMC Medical Director<br>Meeting with Chief Pharmacist<br>(Pharmacy)<br>NTGH<br><br>KCMC Medical Director<br>KCMC Microbiologist<br>KCMC Pharmacists<br>9 – 5pm<br>Pocket Quality Improvement training day<br>(Room 1)<br>Cobalt<br><br>Collection of per diem daily living allowance	Saturday 25th May<br><br>TBC	Sunday 26th May<br><br>TBC	Monday 27th May<br><br>Bank Holiday Monday	Tuesday 28th May<br><br>9:30-11:30<br>KCMC Microbiologist<br>KCMC Pharmacists<br>Bone infection ward round<br>WGH<br><br>14:00 – 17:00<br>KCMC Microbiologist<br>KCMC Pharmacists<br><br>Tour of Pharmacy Aseptic Department<br>WGH	Wednesday 29th May<br><br>9:00-9:30<br>KCMC Pharmacists<br>C Diff MDT<br>(Education centre)<br>NTGH 10<br><br>9:30-12:00<br>Pharmacy speciality board<br>(Room 14)<br>NTGH<br><br>11:00-12:00<br>KCMC Microbiologist<br><br>Lab sensitivity<br>NTGH	Thursday 30th May<br><br>KCMC Medical Director<br>KCMC Microbiologist<br>KCMC Pharmacists<br><br>10:00 – 15:00<br>Healthcare Leadership Training<br>Cobalt

Draft programme: CwPAMS Project: Kilimanjaro Christian Medical Centre (KCMC) team visit to Northumbria Healthcare NHS Trust (NHCT)*
03/05/2019

Friday 31st May<br><br>14:30<br>Transport to Newcastle airport for return flight – departure time 16:25

* Northumbria Healthcare NHS Foundation Trust is an NHS foundation trust which provides hospital and community health services in North Tyneside and hospital, community health and adult social care services in Northumberland. It includes nine hospital sites. Two of which were included in the programme

- North Tyneside General Hospital (NTGH)
- Wansbeck General Hospital (WGH)
